# Supplementary material for: Genomic analyses provide insights into breed-of-origin effects from purebreds on three-way crossbred pigs
Source: PeerJ. 2019 Nov 11;7:e8009. doi: 10.7717/peerj.8009 (PMC6855203; doi:10.7717/peerj.8009)
Supplement: Supplemental Information 1 [file peerj-07-8009-s001.pdf]

Supplementary Figures and Tables

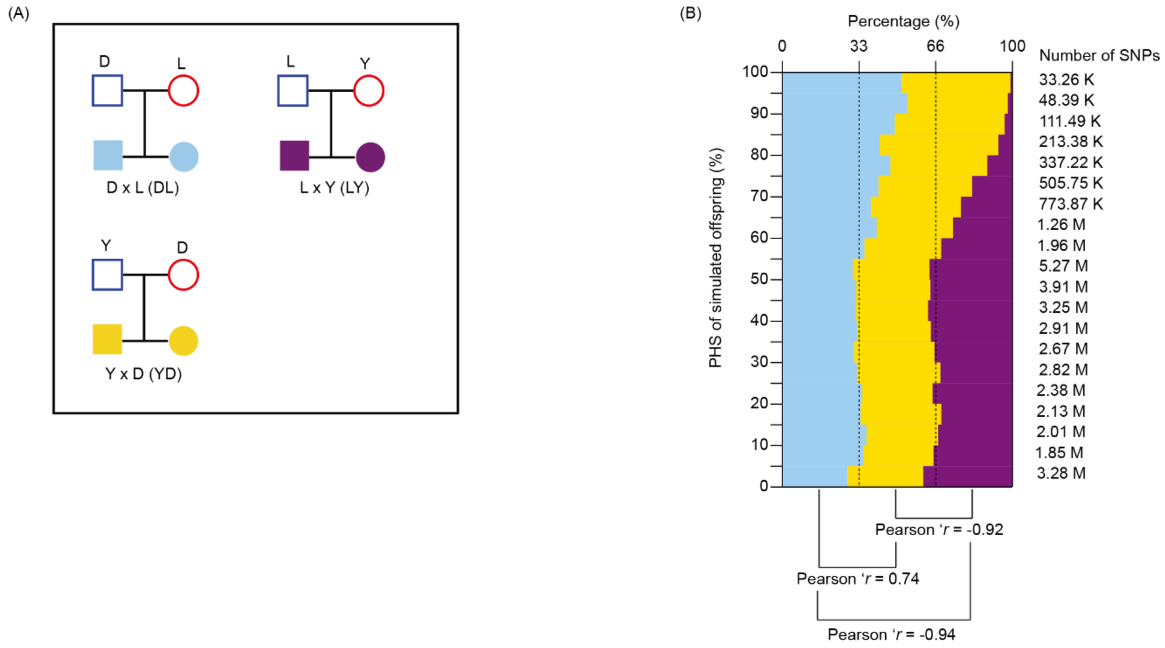

Figure S1. Distribution of heterozygous SNPs in two-way crossbreeding offspring. A Simulation of three kinds of two-way crossbreeding. **B** Distribution of SNP number of the simulated offspring of three kinds of two-way crossbreeding systems for 20 equal intervals (based on PHS, from 0% to 100%, with intervals of 5%). Pearson '*r* was inferred between each two kinds of two-way crossbreeding systems.

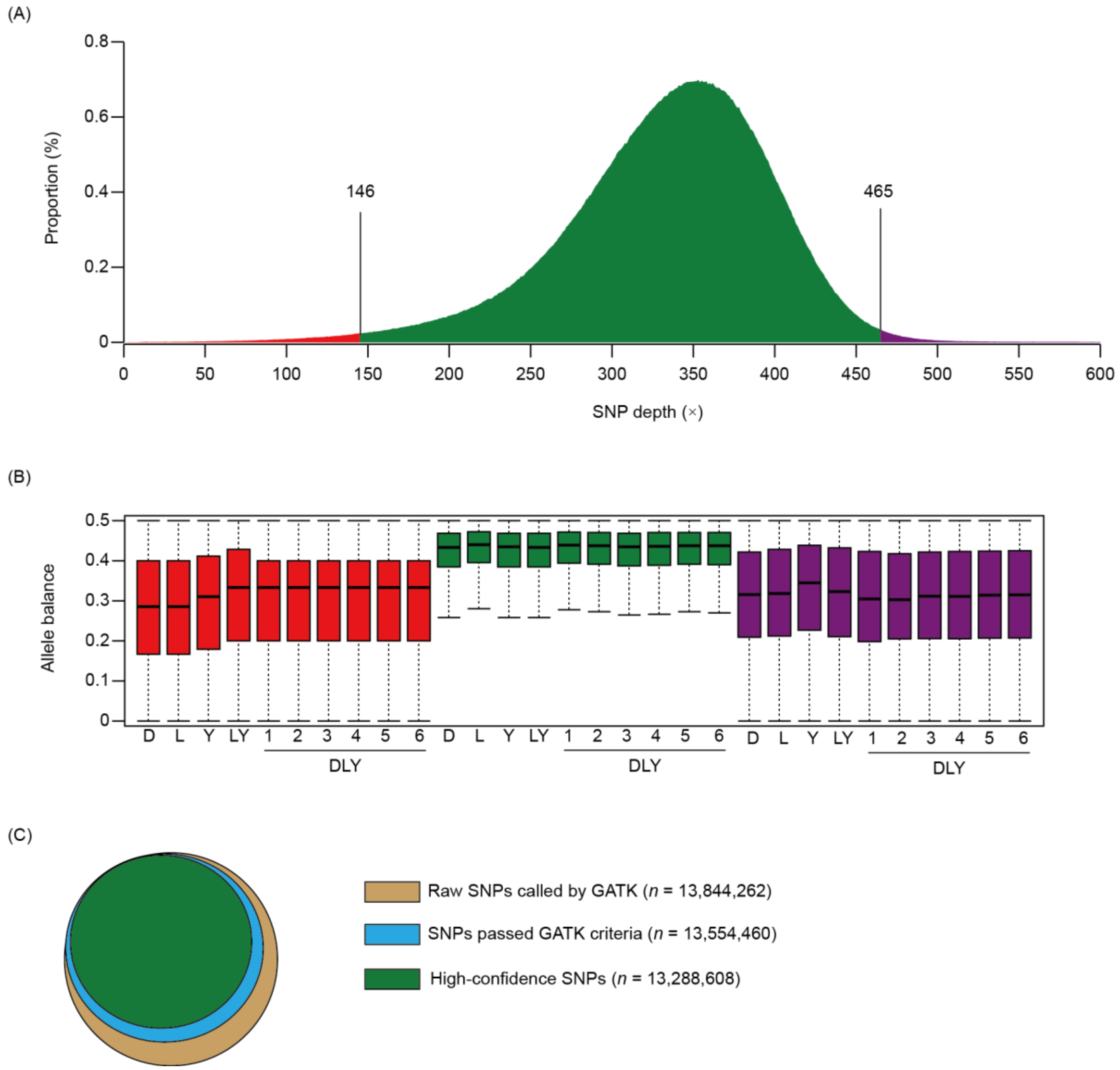

Figure S2. The pipeline of depleting low-confidence SNPs. (A) The distribution of SNP depth of the sequenced DLY pig family. We empirically depleted ~1% SNPs located in left tail (SNP depth < 146, marked in red) and right tail (SNP depth > 465, marked in purple). **(B)** The distribution of allele balance for left tail SNPs (red), high-confidence SNPs (green) and right tail SNPs (purple) of ten sequenced pigs. **(C)** The number of raw SNPs called by GATK (n = 13,844,262), SNPs passed GATK criteria (n = 13,554,460) and high-confidence SNPs (n = 13,288,608).

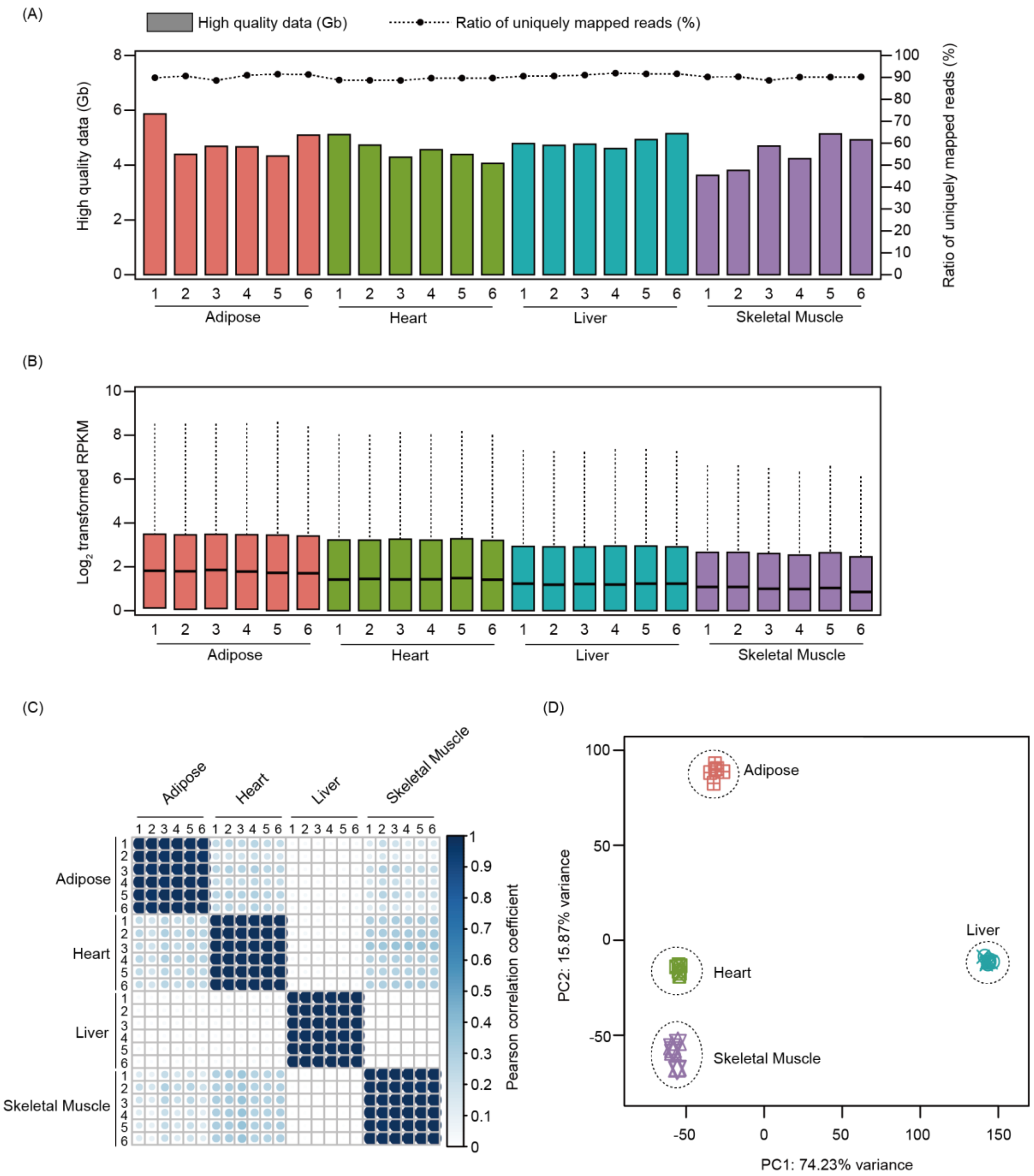

Figure S3. Summary of RNA-seq for adipose, liver, heart and skeletal muscle. The statistics of high-quality data and uniquely mapped reads ratio **(A)**, **t**he log_2 transformed RPKM distribution **(B)**, the pearson correlation coefficient **(C)** and PCA result **(D)** for adipose, heart, liver and skeletal muscle across 24 DLY individuals were shown.

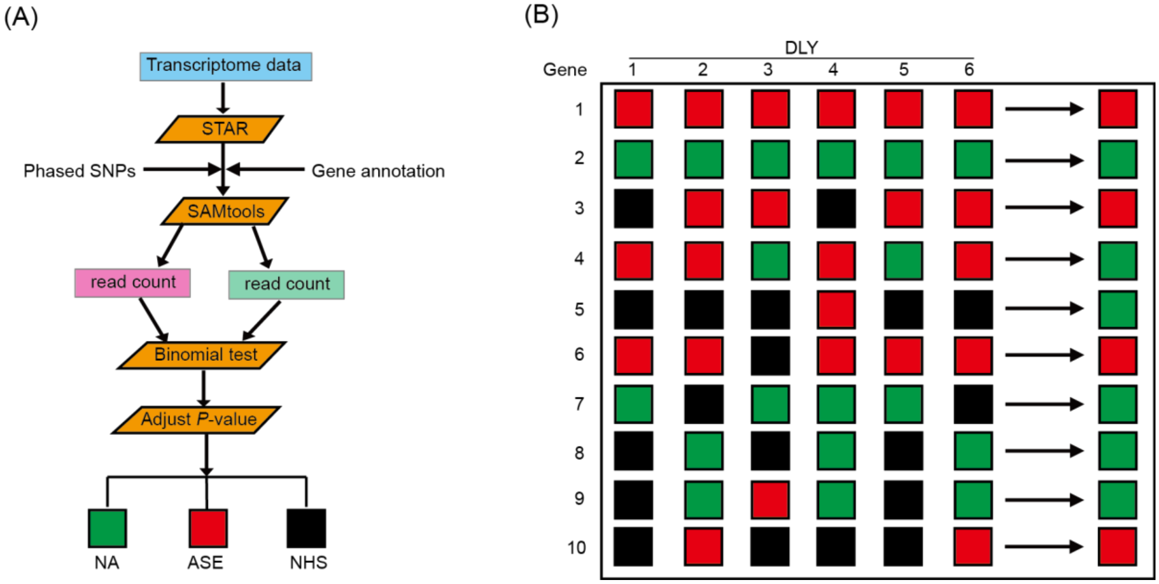

Figure S4. The pipeline of ASE genes identification. (A) The process of mapping, binomial test and classification. Transcriptome data were mapped with STAR and the total number of reads for each gene aligning to different alleles were counted by SAMtools. Each gene was classified to three categories, including ASE (adjust-P < 0.05, binomial test), NA (not significant for allele-specific expression) and NHS (no heterozygous SNP). **(B)** The principle of ASE identification: more than two DLY individuals harbor phased exonic SNPs and are all ASE categories.

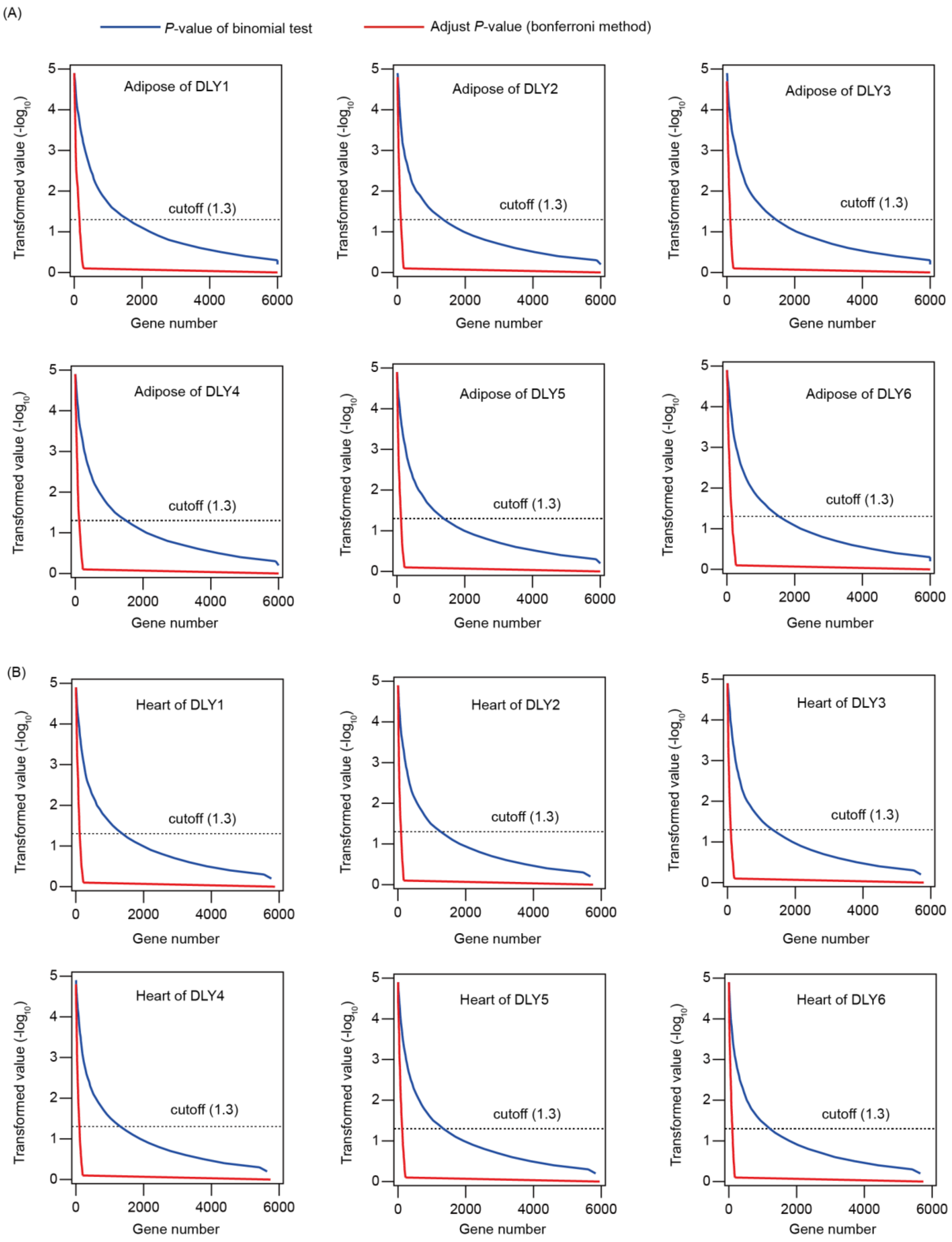
(A)
P-value of binomial test
Adjust P-value (bonferroni method)
Adipose of DLY1
Adipose of DLY2
Adipose of DLY3
Adipose of DLY4
Adipose of DLY5
Adipose of DLY6
(B)
Heart of DLY1
Heart of DLY2
Heart of DLY3
Heart of DLY4
Heart of DLY5
Heart of DLY6
cutoff (1.3)
Transformed value (-log10)
Gene number
0
1
2
3
4
5
0
2000
4000
6000

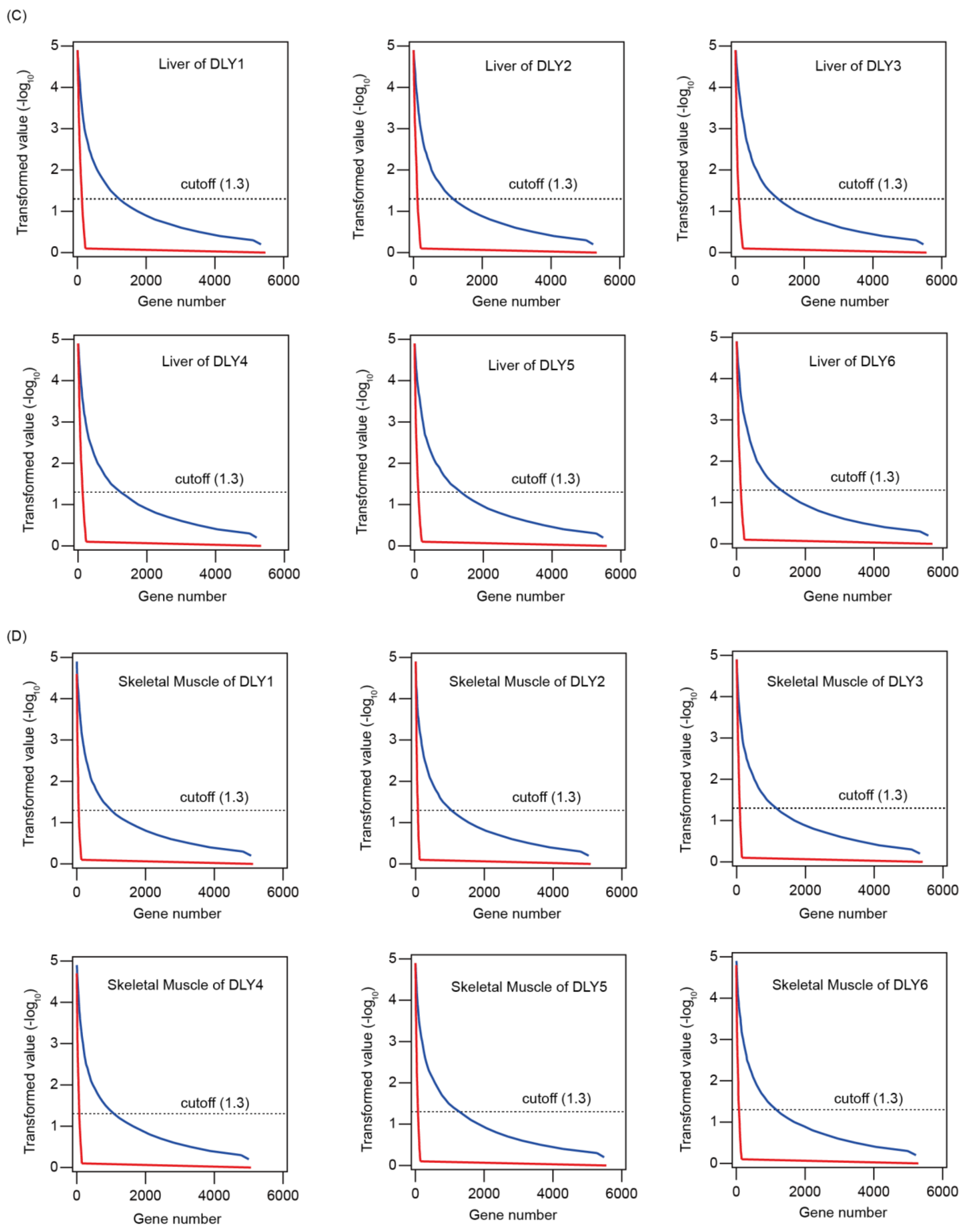

Figure S5. *P*-value adjustment using Bonferroni method. The *P*-values calculated by binomial test were adjusted using bonferroni method for adipose **(A)**, heart **(B)**, liver **(C)** and skeletal muscle **(D)**.

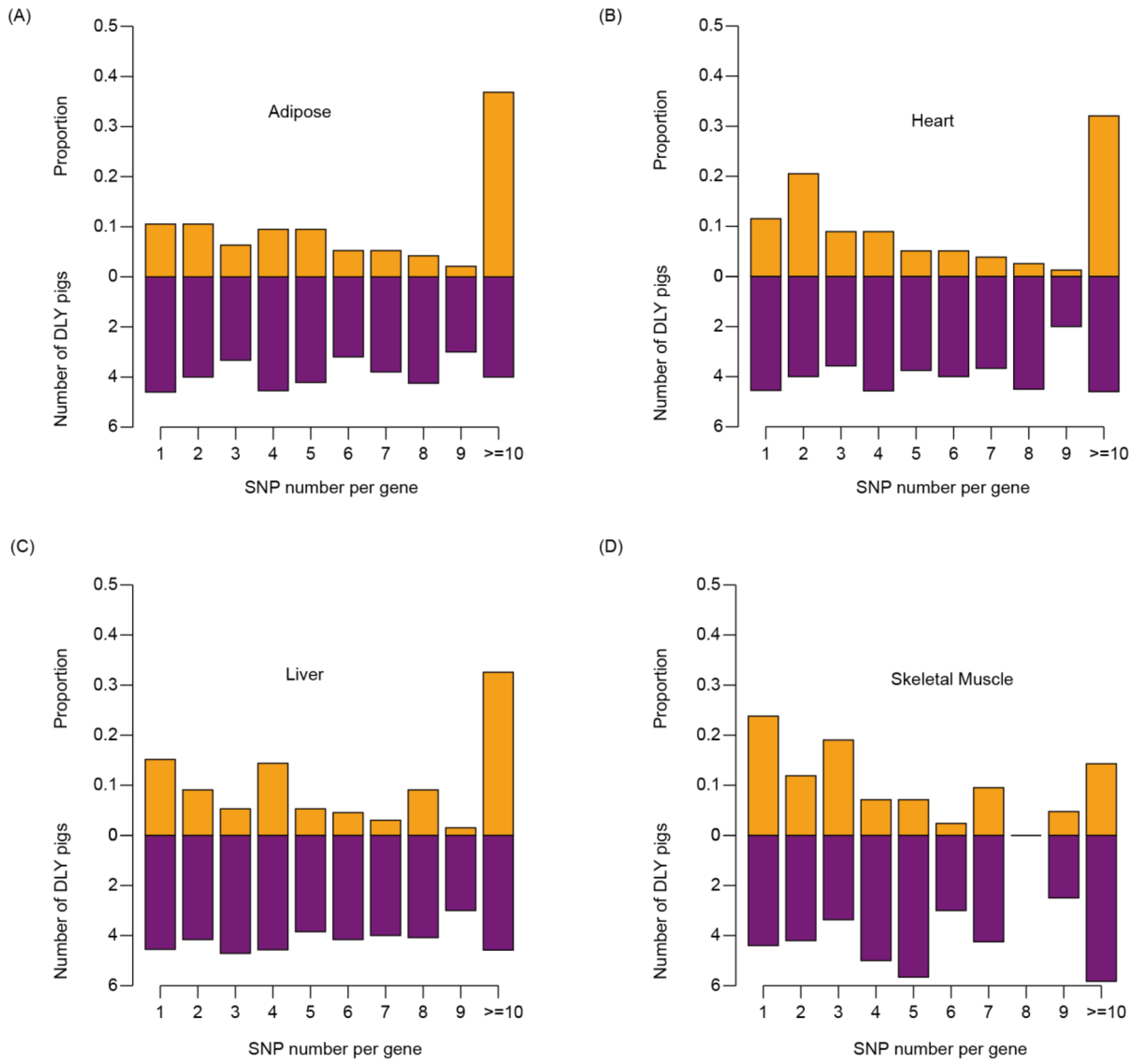

Figure S6. The Statistics of SNP number for ASE genes. For each ASE gene, the proportion (marked in orange) and the number of DLY pigs (marked in purple) for each rank of average SNP number (keep integer) were calculated for adipose **(A)**, heart **(B)**, liver **(C)** and skeletal muscle **(D)**.

>Ensembl GeneID: ENSSSCG00000004834
>Gene Symbol: ***NDN***
>Tissue of ASE pattern: Adipose, Heart, Liver, Skeletal Muscle (Known imprinted gene)

SNP locus: Chr1: 142412685

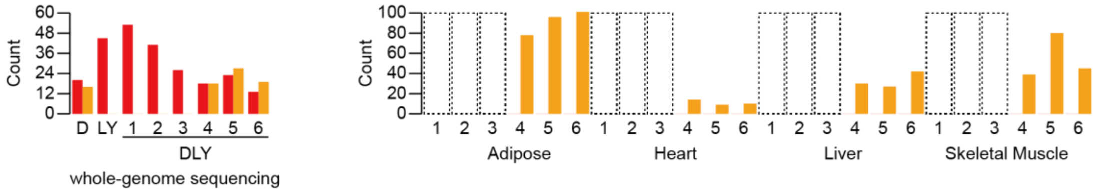

>Ensembl GeneID: ENSSSCG00000037158
>Gene Symbol: ***MTURN***
>Tissue of ASE pattern: Adipose

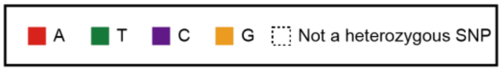

SNP locus: Chr18: 42801778

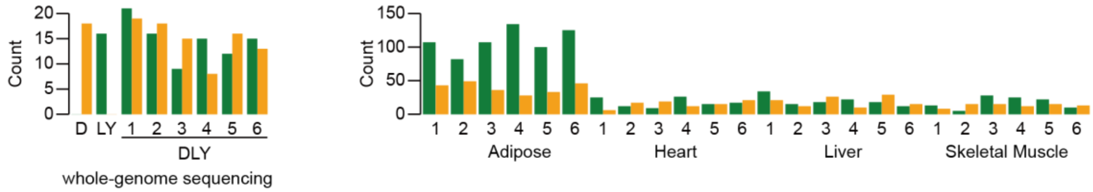

>Ensembl GeneID: ENSSSCG00000038399
>Gene Symbol: ***ATP5PF***
>Tissue of ASE pattern: Adipose, Heart, Skeletal Muscle

SNP locus: Chr13: 189281509

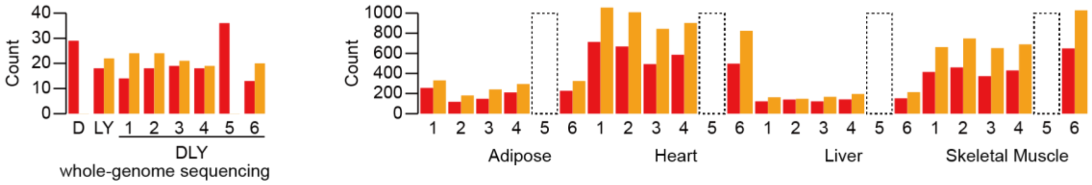

>Ensembl GeneID: ENSSSCG00000003465
>Gene Symbol: ***FBLIM1***
>Tissue of ASE pattern: Adipose

SNP locus: Chr6: 74961704

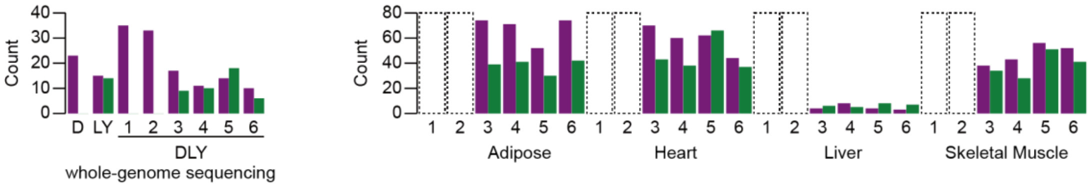

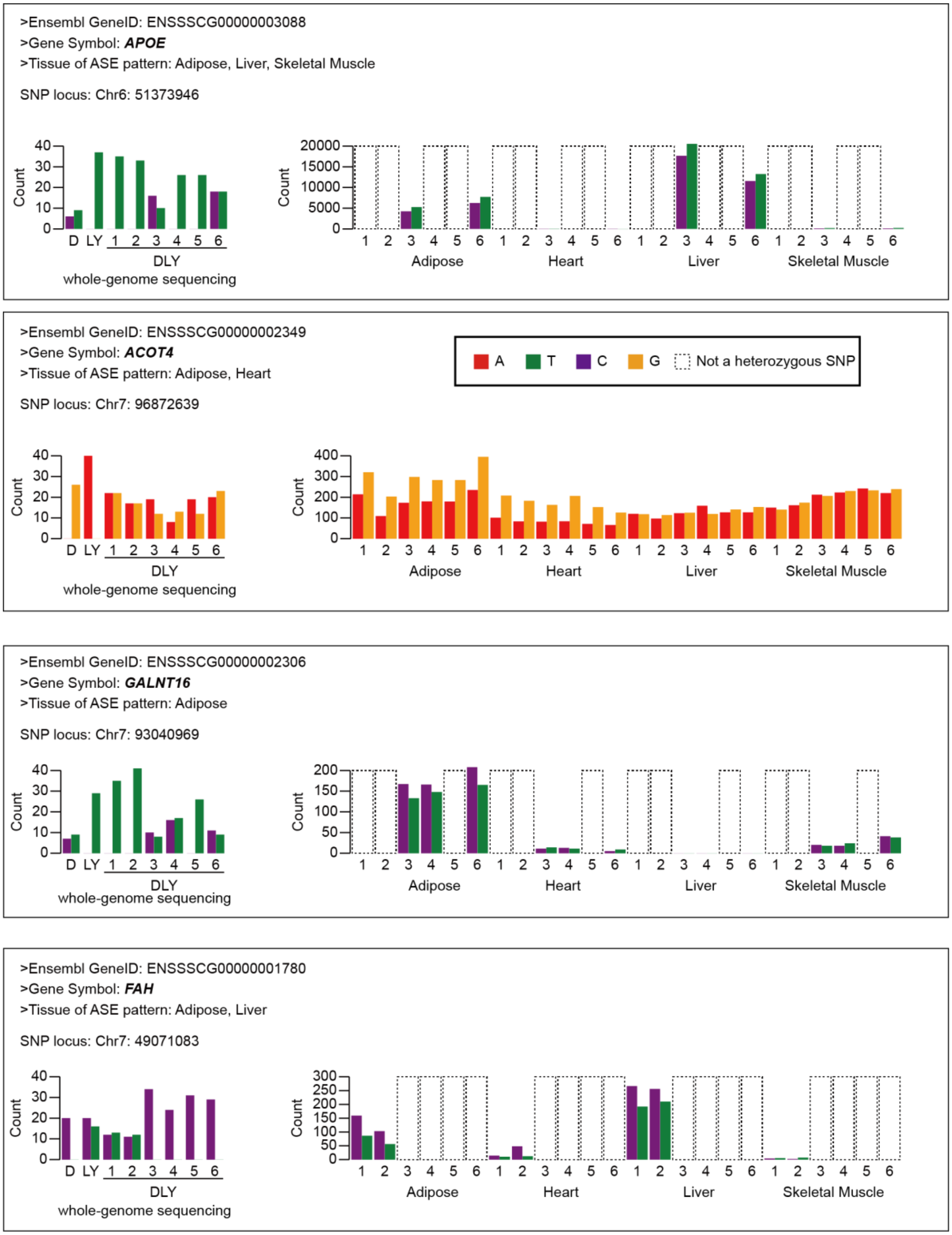
>Ensembl GeneID: ENSSSCG00000003088
>Gene Symbol: APOE
>Tissue of ASE pattern: Adipose, Liver, Skeletal Muscle
SNP locus: Chr6: 51373946
Count
D LY 1 2 3 4 5 6
DLY
whole-genome sequencing
Adipose
Heart
Liver
Skeletal Muscle
>Ensembl GeneID: ENSSSCG00000002349
>Gene Symbol: ACOT4
>Tissue of ASE pattern: Adipose, Heart
SNP locus: Chr7: 96872639
A T C G Not a heterozygous SNP
>Ensembl GeneID: ENSSSCG00000002306
>Gene Symbol: GALNT16
>Tissue of ASE pattern: Adipose
SNP locus: Chr7: 93040969
>Ensembl GeneID: ENSSSCG00000001780
>Gene Symbol: FAH
>Tissue of ASE pattern: Adipose, Liver
SNP locus: Chr7: 49071083

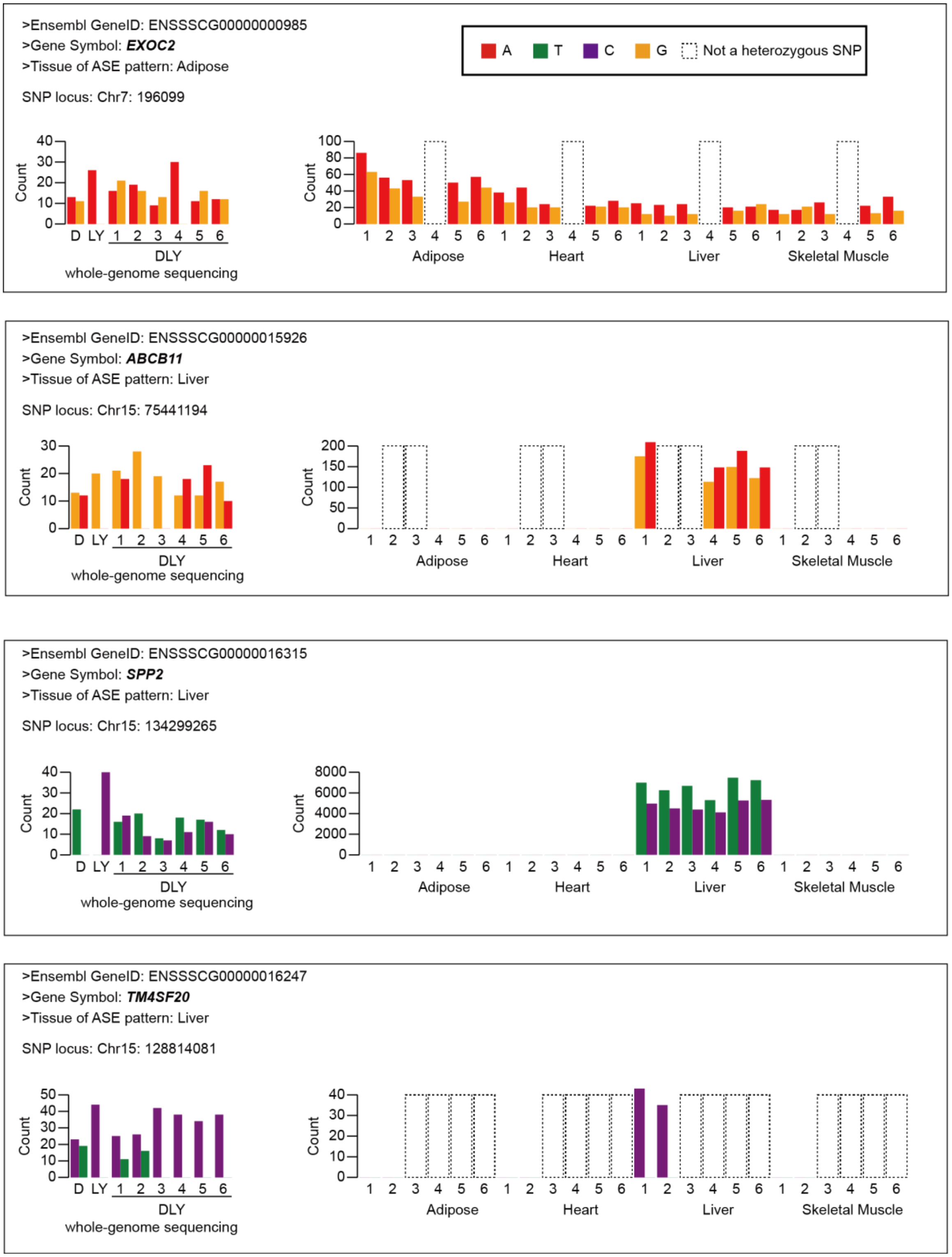

>Ensembl GeneID: ENSSSCG00000000985
>Gene Symbol: EXOC2
>Tissue of ASE pattern: Adipose
SNP locus: Chr7: 196099
A
T
C
G
Not a heterozygous SNP
Count
D
LY
DLY
whole-genome sequencing
Adipose
Heart
Liver
Skeletal Muscle
>Ensembl GeneID: ENSSSCG00000015926
>Gene Symbol: ABCB11
>Tissue of ASE pattern: Liver
SNP locus: Chr15: 75441194
>Ensembl GeneID: ENSSSCG00000016315
>Gene Symbol: SPP2
>Tissue of ASE pattern: Liver
SNP locus: Chr15: 134299265
>Ensembl GeneID: ENSSSCG00000016247
>Gene Symbol: TM4SF20
>Tissue of ASE pattern: Liver
SNP locus: Chr15: 128814081

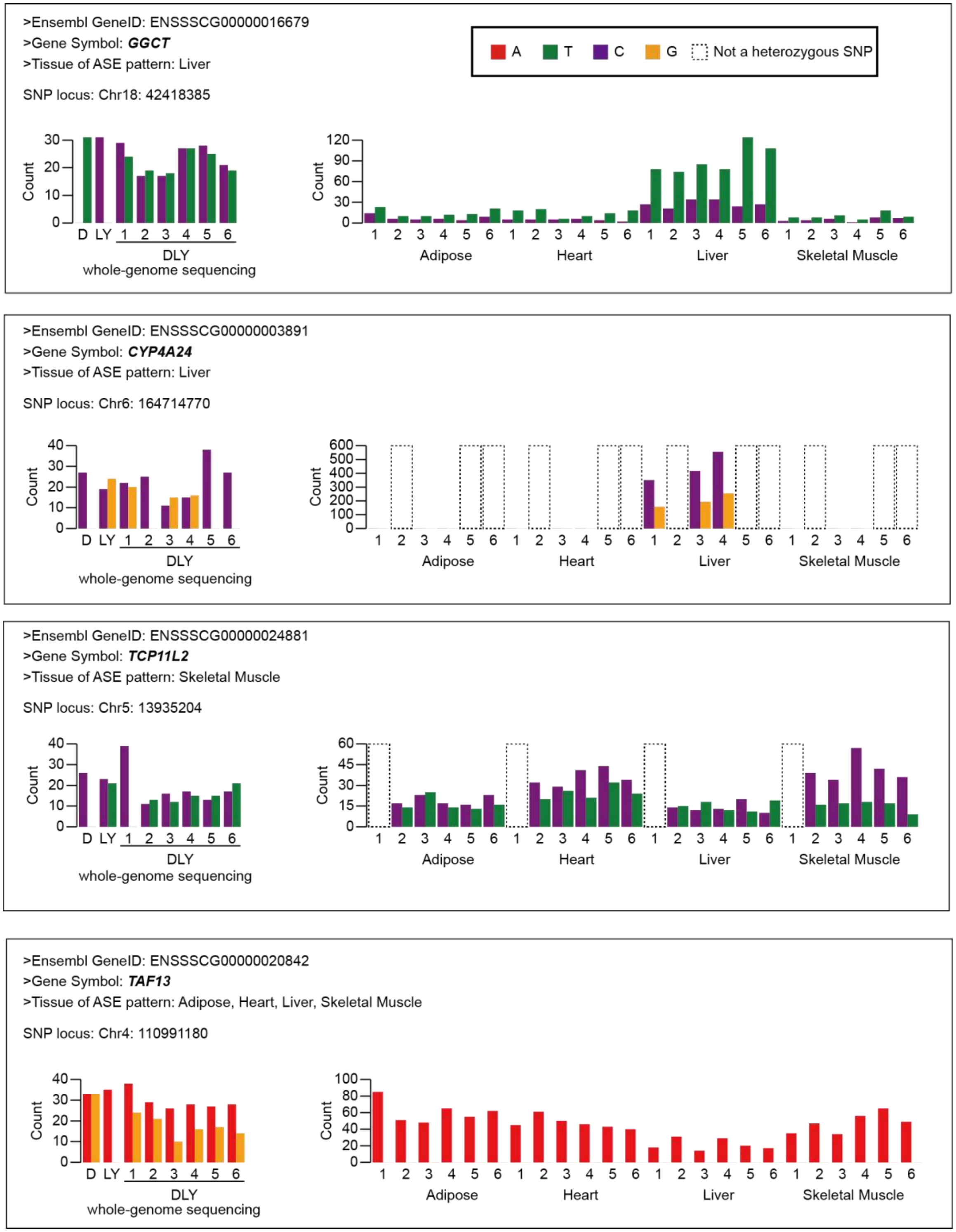
>Ensembl GeneID: ENSSSCG00000016679
>Gene Symbol: GGCT
>Tissue of ASE pattern: Liver
SNP locus: Chr18: 42418385
A
T
C
G
Not a heterozygous SNP
Count
D LY 1 2 3 4 5 6
DLY
whole-genome sequencing
Adipose
Heart
Liver
Skeletal Muscle
>Ensembl GeneID: ENSSSCG00000003891
>Gene Symbol: CYP4A24
>Tissue of ASE pattern: Liver
SNP locus: Chr6: 164714770
Count
D LY 1 2 3 4 5 6
DLY
whole-genome sequencing
Adipose
Heart
Liver
Skeletal Muscle
>Ensembl GeneID: ENSSSCG00000024881
>Gene Symbol: TCP11L2
>Tissue of ASE pattern: Skeletal Muscle
SNP locus: Chr5: 13935204
Count
D LY 1 2 3 4 5 6
DLY
whole-genome sequencing
Adipose
Heart
Liver
Skeletal Muscle
>Ensembl GeneID: ENSSSCG00000020842
>Gene Symbol: TAF13
>Tissue of ASE pattern: Adipose, Heart, Liver, Skeletal Muscle
SNP locus: Chr4: 110991180
Count
D LY 1 2 3 4 5 6
DLY
whole-genome sequencing
Adipose
Heart
Liver
Skeletal Muscle

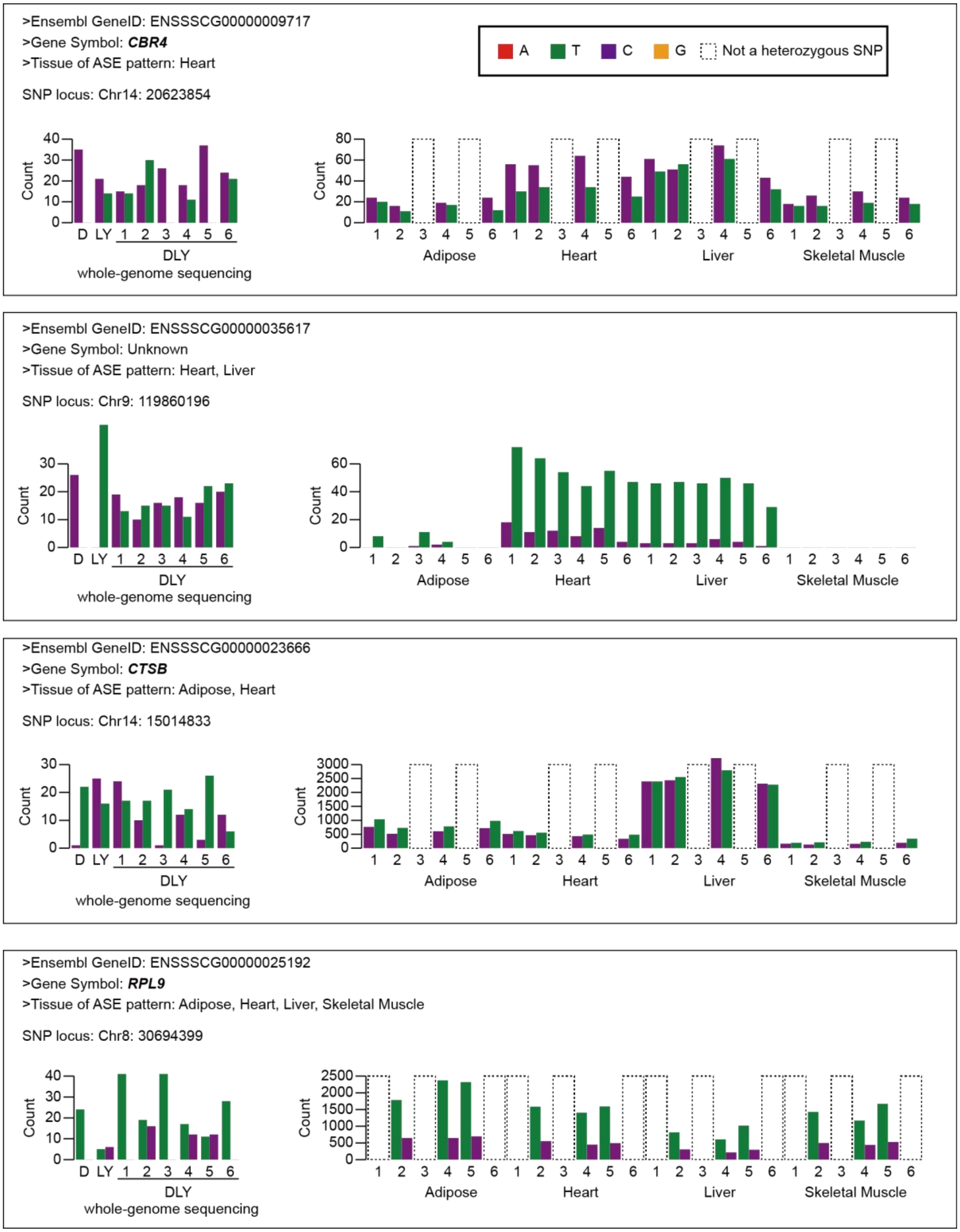

>Ensembl GeneID: ENSSSCG00000009717
>Gene Symbol: CBR4
>Tissue of ASE pattern: Heart
SNP locus: Chr14: 20623854
A
T
C
G
Not a heterozygous SNP
Count
D LY 1 2 3 4 5 6
DLY
whole-genome sequencing
1 2 3 4 5 6 1 2 3 4 5 6 1 2 3 4 5 6 1 2 3 4 5 6
Adipose
Heart
Liver
Skeletal Muscle
>Ensembl GeneID: ENSSSCG00000035617
>Gene Symbol: Unknown
>Tissue of ASE pattern: Heart, Liver
SNP locus: Chr9: 119860196
>Ensembl GeneID: ENSSSCG00000023666
>Gene Symbol: CTSB
>Tissue of ASE pattern: Adipose, Heart
SNP locus: Chr14: 15014833
>Ensembl GeneID: ENSSSCG00000025192
>Gene Symbol: RPL9
>Tissue of ASE pattern: Adipose, Heart, Liver, Skeletal Muscle
SNP locus: Chr8: 30694399

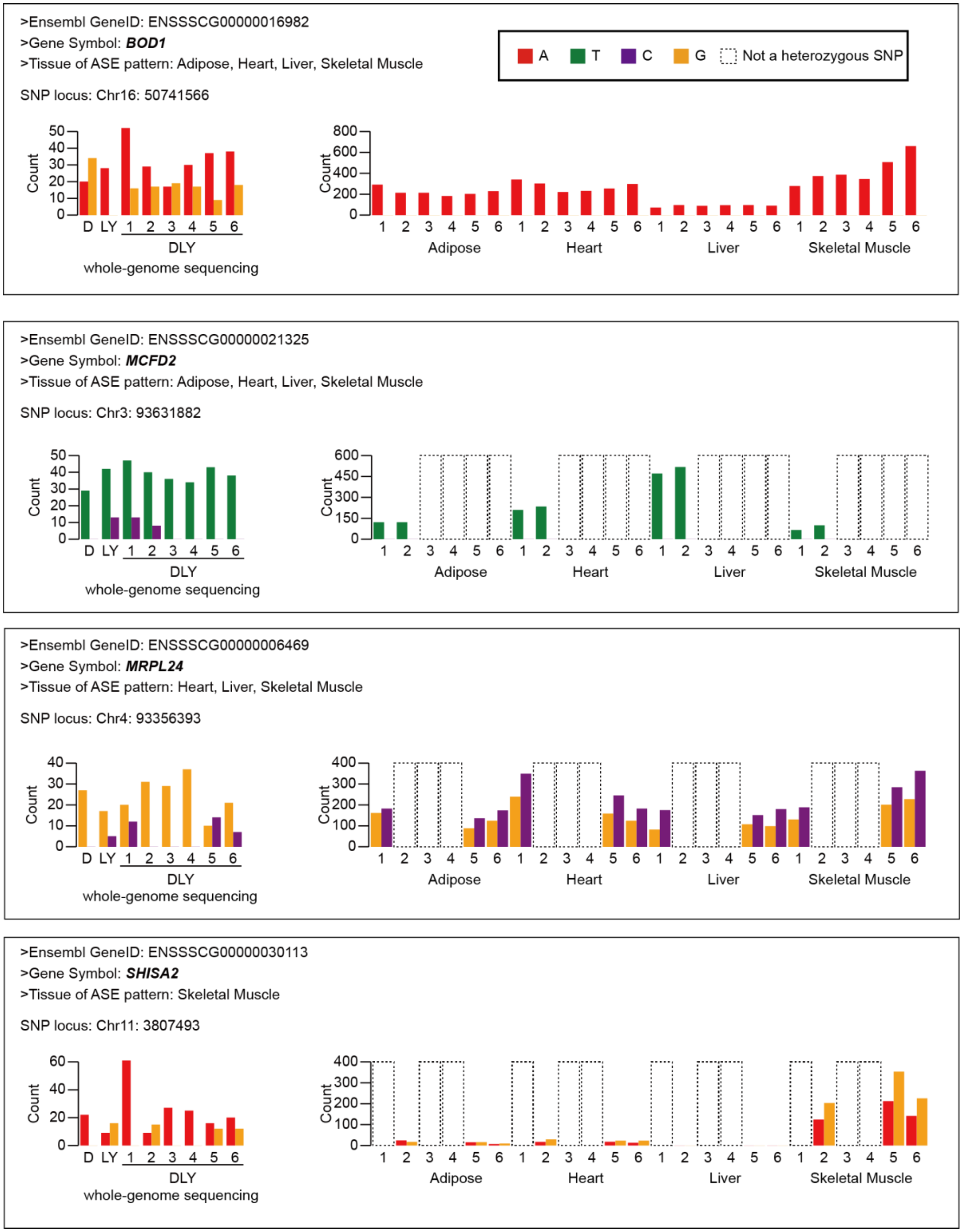
>Ensembl GeneID: ENSSSCG00000016982
>Gene Symbol: BOD1
>Tissue of ASE pattern: Adipose, Heart, Liver, Skeletal Muscle
SNP locus: Chr16: 50741566
A
T
C
G
Not a heterozygous SNP
Count
D LY 1 2 3 4 5 6
DLY
whole-genome sequencing
Adipose
Heart
Liver
Skeletal Muscle
>Ensembl GeneID: ENSSSCG00000021325
>Gene Symbol: MCFD2
>Tissue of ASE pattern: Adipose, Heart, Liver, Skeletal Muscle
SNP locus: Chr3: 93631882
Count
DLY
whole-genome sequencing
Adipose
Heart
Liver
Skeletal Muscle
>Ensembl GeneID: ENSSSCG00000006469
>Gene Symbol: MRPL24
>Tissue of ASE pattern: Heart, Liver, Skeletal Muscle
SNP locus: Chr4: 93356393
Count
DLY
whole-genome sequencing
Adipose
Heart
Liver
Skeletal Muscle
>Ensembl GeneID: ENSSSCG00000030113
>Gene Symbol: SHISA2
>Tissue of ASE pattern: Skeletal Muscle
SNP locus: Chr11: 3807493
Count
DLY
whole-genome sequencing
Adipose
Heart
Liver
Skeletal Muscle

>Ensembl GeneID: ENSSSCG00000035293
>Gene Symbol: ***IGF2***
>Tissue of ASE pattern: Adipose, Heart, Liver, Skeletal Muscle (Known imprinted gene)

SNP locus: Chr2: 1469990

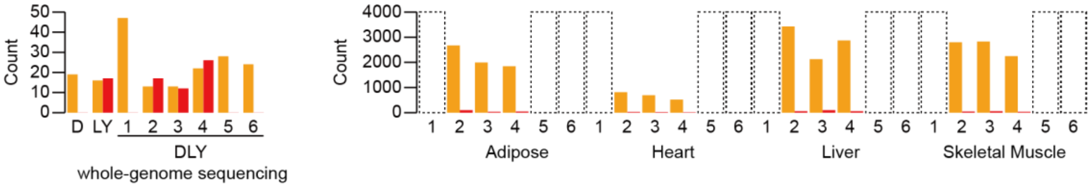

>Ensembl GeneID: ENSSSCG00000014075
>Gene Symbol: ***NSA2***
>Tissue of ASE pattern: Adipose, Heart, Liver, Skeletal Muscle

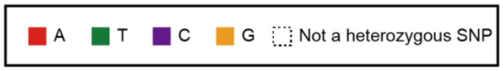

SNP locus: Chr2: 83847997

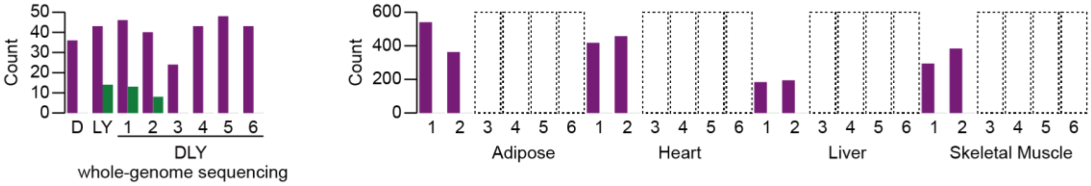

>Ensembl GeneID: ENSSSCG00000033727
>Gene Symbol: ***GPX1***
>Tissue of ASE pattern: Adipose, Heart, Liver, Skeletal Muscle

SNP locus: Chr13: 31917336

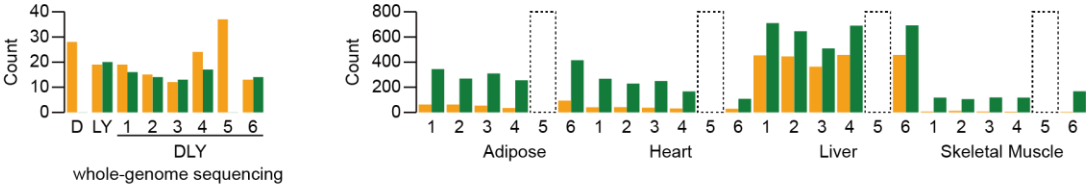

>Ensembl GeneID: ENSSSCG00000035223
>Gene Symbol: Unknown
>Tissue of ASE pattern: Skeletal Muscle

SNP locus: Chr1: 137879514

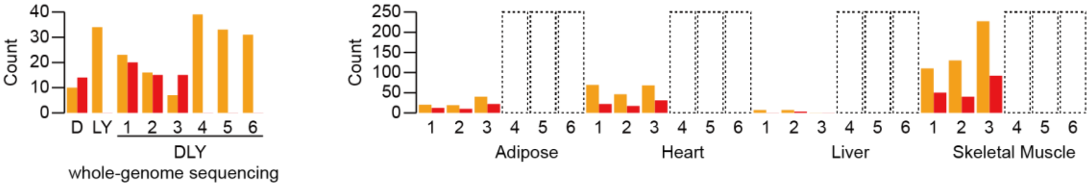

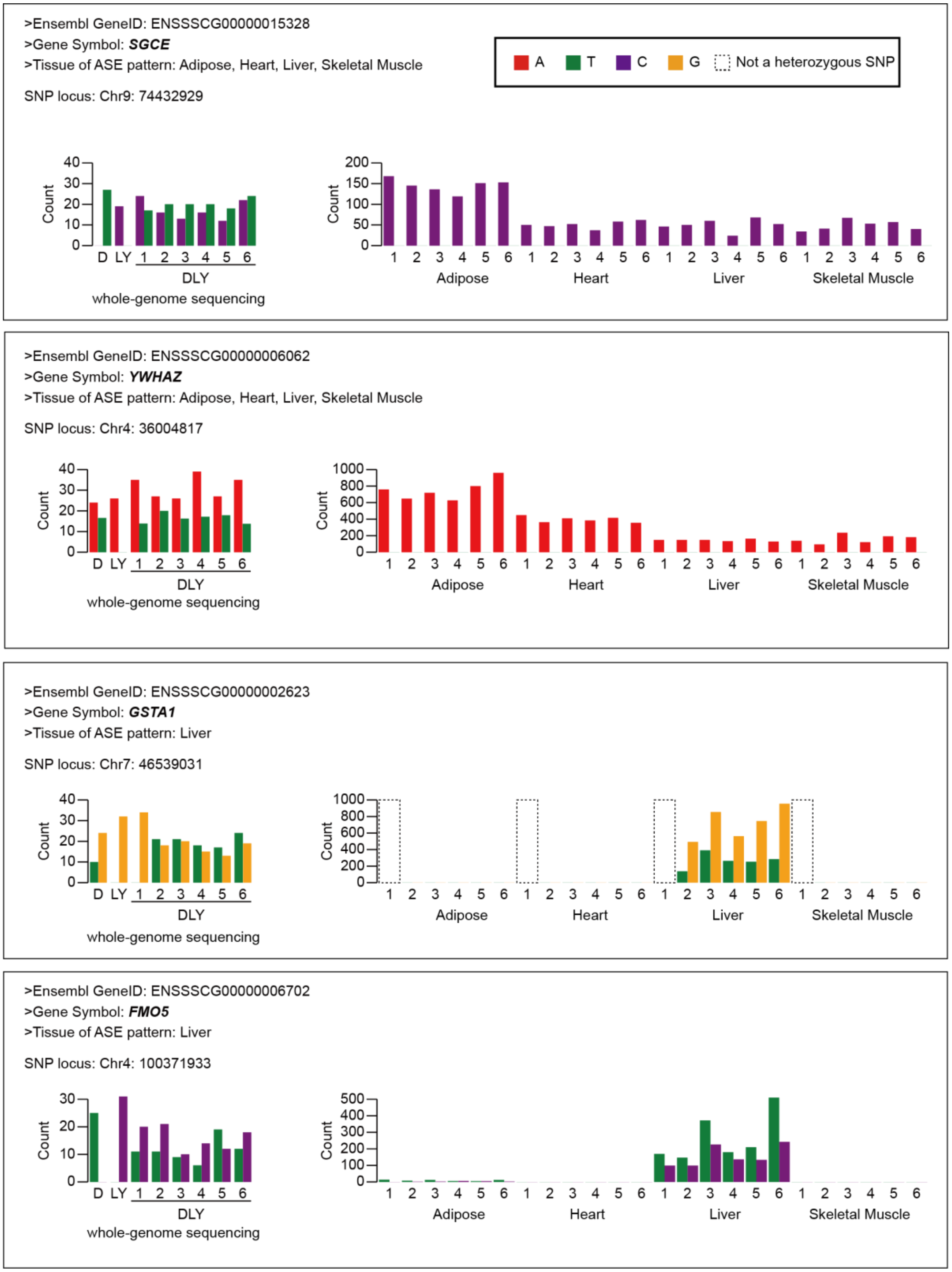

>Ensembl GeneID: ENSSSCG00000015328
>Gene Symbol: ***SGCE***
>Tissue of ASE pattern: Adipose, Heart, Liver, Skeletal Muscle
SNP locus: Chr9: 74432929
A
T
C
G
Not a heterozygous SNP
Count
D LY 1 2 3 4 5 6
DLY
whole-genome sequencing
Adipose
Heart
Liver
Skeletal Muscle
>Ensembl GeneID: ENSSSCG00000006062
>Gene Symbol: ***YWHAZ***
>Tissue of ASE pattern: Adipose, Heart, Liver, Skeletal Muscle
SNP locus: Chr4: 36004817
>Ensembl GeneID: ENSSSCG00000002623
>Gene Symbol: ***GSTA1***
>Tissue of ASE pattern: Liver
SNP locus: Chr7: 46539031
>Ensembl GeneID: ENSSSCG00000006702
>Gene Symbol: ***FMO5***
>Tissue of ASE pattern: Liver
SNP locus: Chr4: 100371933

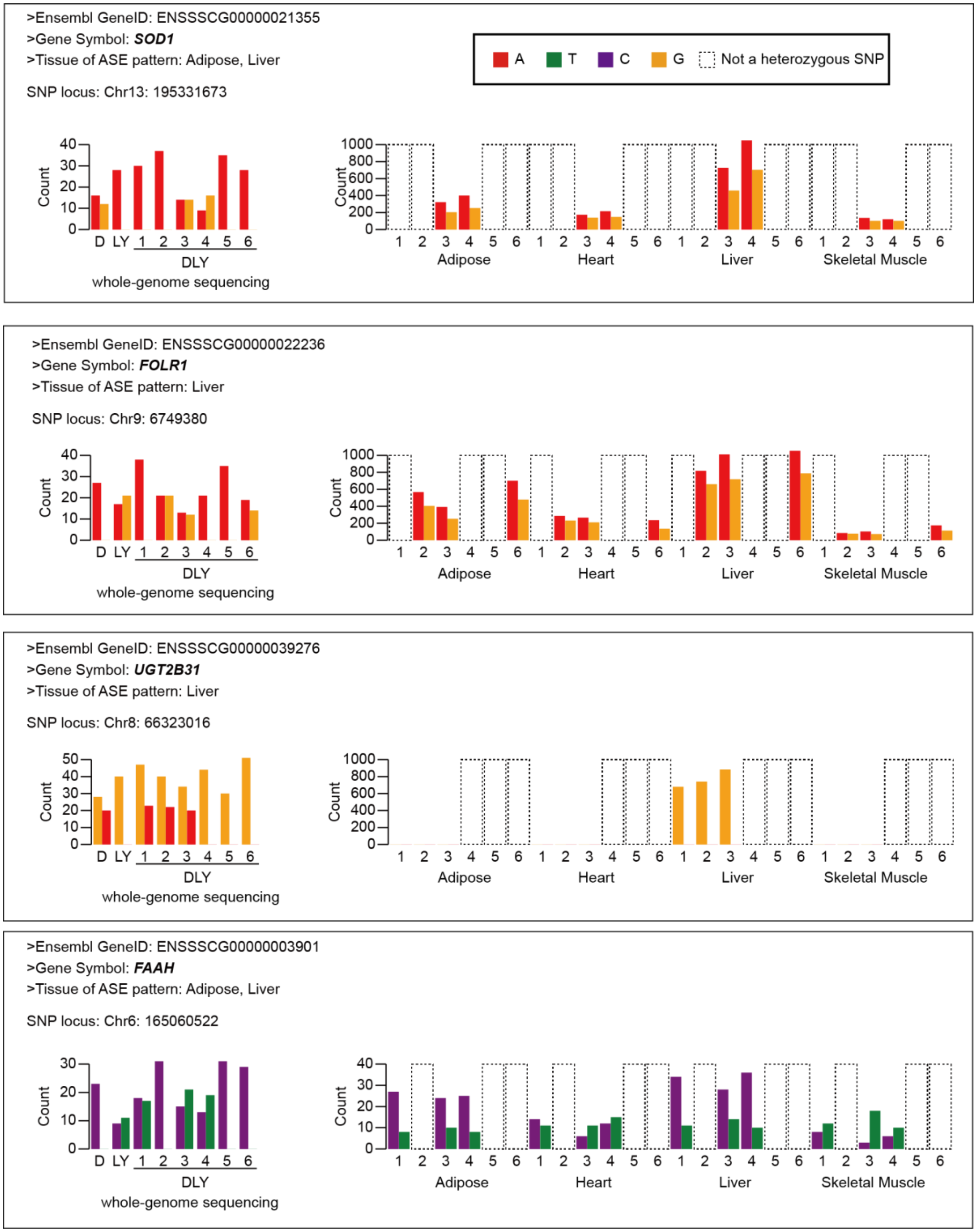
>Ensembl GeneID: ENSSSCG00000021355
>Gene Symbol: ***SOD1***
>Tissue of ASE pattern: Adipose, Liver
SNP locus: Chr13: 195331673
A
T
C
G
Not a heterozygous SNP
Count
D LY 1 2 3 4 5 6
DLY
whole-genome sequencing
Adipose
Heart
Liver
Skeletal Muscle
>Ensembl GeneID: ENSSSCG00000022236
>Gene Symbol: ***FOLR1***
>Tissue of ASE pattern: Liver
SNP locus: Chr9: 6749380
>Ensembl GeneID: ENSSSCG00000039276
>Gene Symbol: ***UGT2B31***
>Tissue of ASE pattern: Liver
SNP locus: Chr8: 66323016
>Ensembl GeneID: ENSSSCG00000003901
>Gene Symbol: ***FAAH***
>Tissue of ASE pattern: Adipose, Liver
SNP locus: Chr6: 165060522

>Ensembl GeneID: ENSSSCG00000001849
>Gene Symbol: ***ANPEP***
>Tissue of ASE pattern: Liver

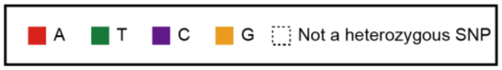

SNP locus: Chr7: 55360022

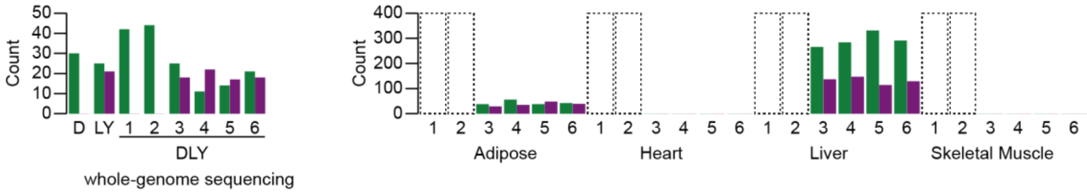

>Ensembl GeneID: ENSSSCG00000000576
>Gene Symbol: ***LDHB***
>Tissue of ASE pattern: Adipose, Heart, Liver, Skeletal Muscle

SNP locus: Chr5: 51825042

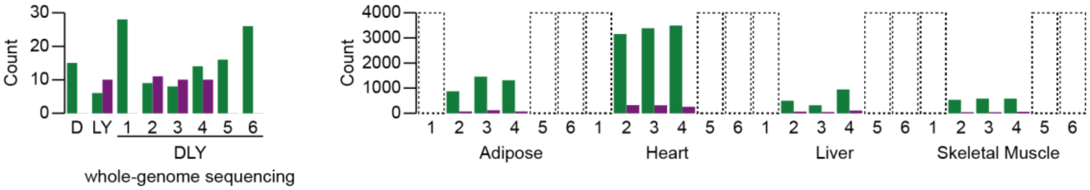

>Ensembl GeneID: ENSSSCG00000000046
>Gene Symbol: ***CYP2D25***
>Tissue of ASE pattern: Liver

SNP locus: Chr5: 6542289

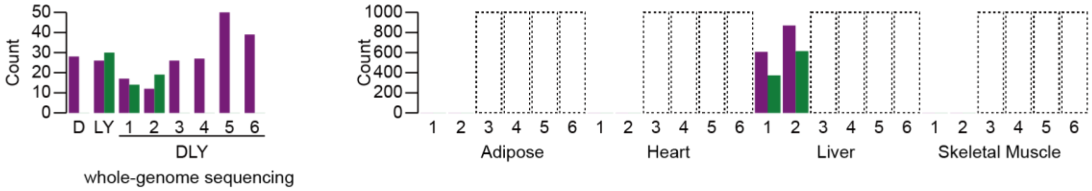

>Ensembl GeneID: ENSSSCG00000002351
>Gene Symbol: ***PTGR2***
>Tissue of ASE pattern: Liver

SNP locus: Chr7: 97188626

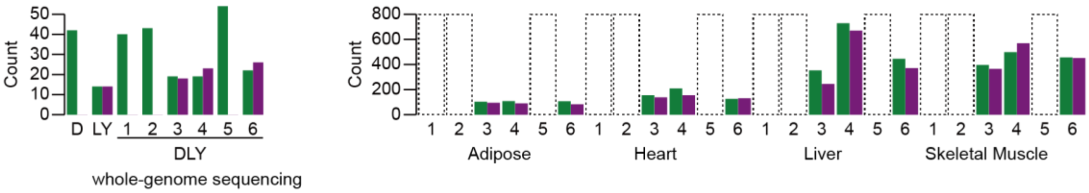

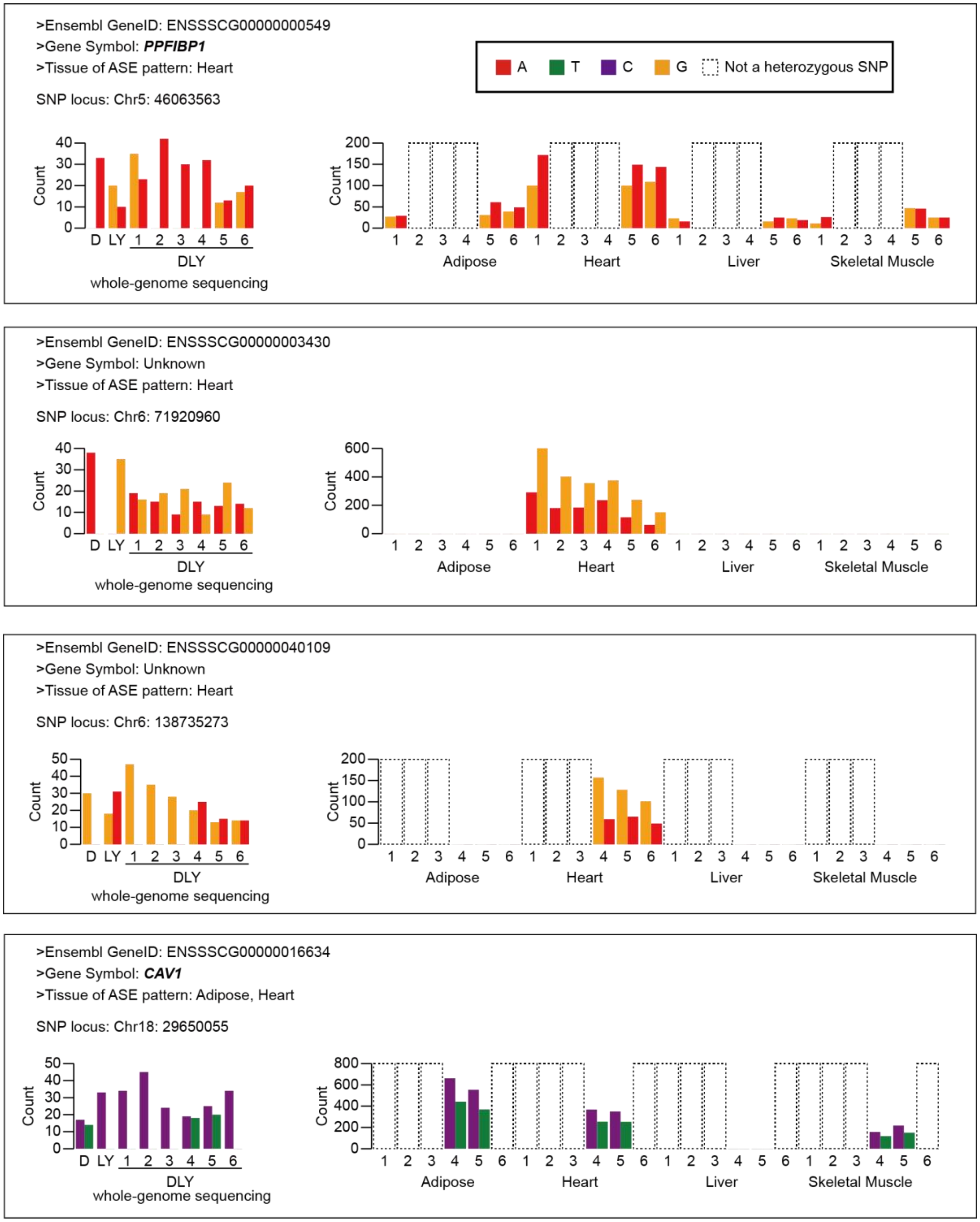

>Ensembl GeneID: ENSSSCG00000000549
>Gene Symbol: ***PPFIBP1***
>Tissue of ASE pattern: Heart
SNP locus: Chr5: 46063563
A
T
C
G
Not a heterozygous SNP
Count
D LY 1 2 3 4 5 6
DLY
whole-genome sequencing
Adipose
Heart
Liver
Skeletal Muscle
>Ensembl GeneID: ENSSSCG00000003430
>Gene Symbol: Unknown
>Tissue of ASE pattern: Heart
SNP locus: Chr6: 71920960
>Ensembl GeneID: ENSSSCG00000040109
>Gene Symbol: Unknown
>Tissue of ASE pattern: Heart
SNP locus: Chr6: 138735273
>Ensembl GeneID: ENSSSCG00000016634
>Gene Symbol: ***CAV1***
>Tissue of ASE pattern: Adipose, Heart
SNP locus: Chr18: 29650055

>Ensembl GeneID: ENSSSCG00000011827
>Gene Symbol: ***LSG1***
>Tissue of ASE pattern: Adipose, Heart, Liver

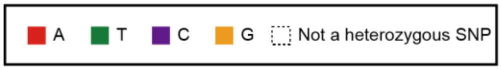

SNP locus: Chr13: 131629386

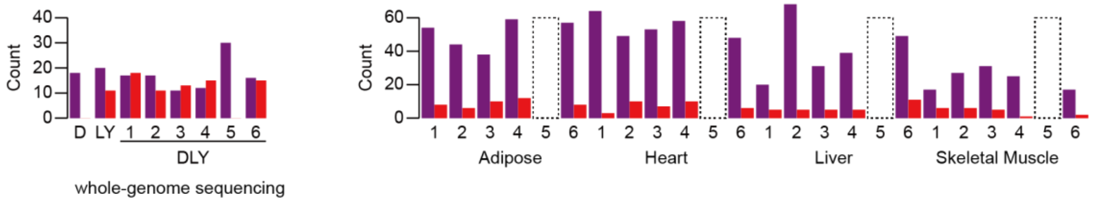

>Ensembl GeneID: ENSSSCG00000016714
>Gene Symbol: ***CYCS***
>Tissue of ASE pattern: Heart

SNP locus: Chr18: 47169958

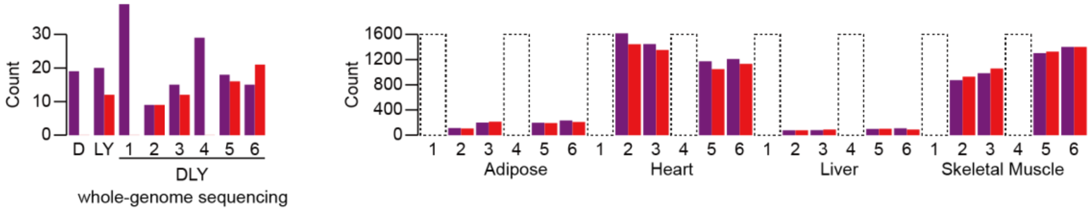

>Ensembl GeneID: ENSSSCG00000003691
>Gene Symbol: ***MRLC2***
>Tissue of ASE pattern: Heart, Skeletal Muscle

SNP locus: Chr6: 103391852

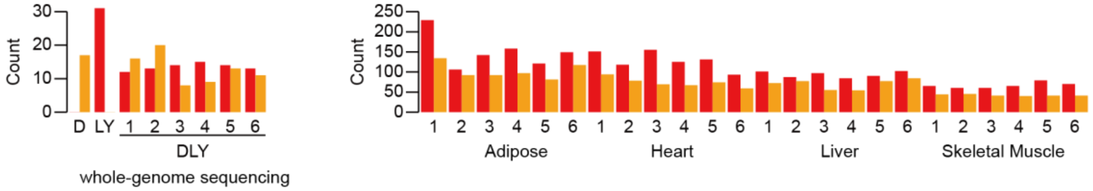

>Ensembl GeneID: ENSSSCG00000034663
>Gene Symbol: Unknown
>Tissue of ASE pattern: Adipose, Heart, Liver, Skeletal Muscle

SNP locus: Chr4: 36008105

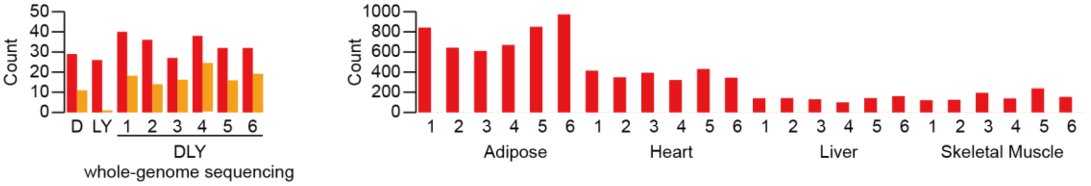

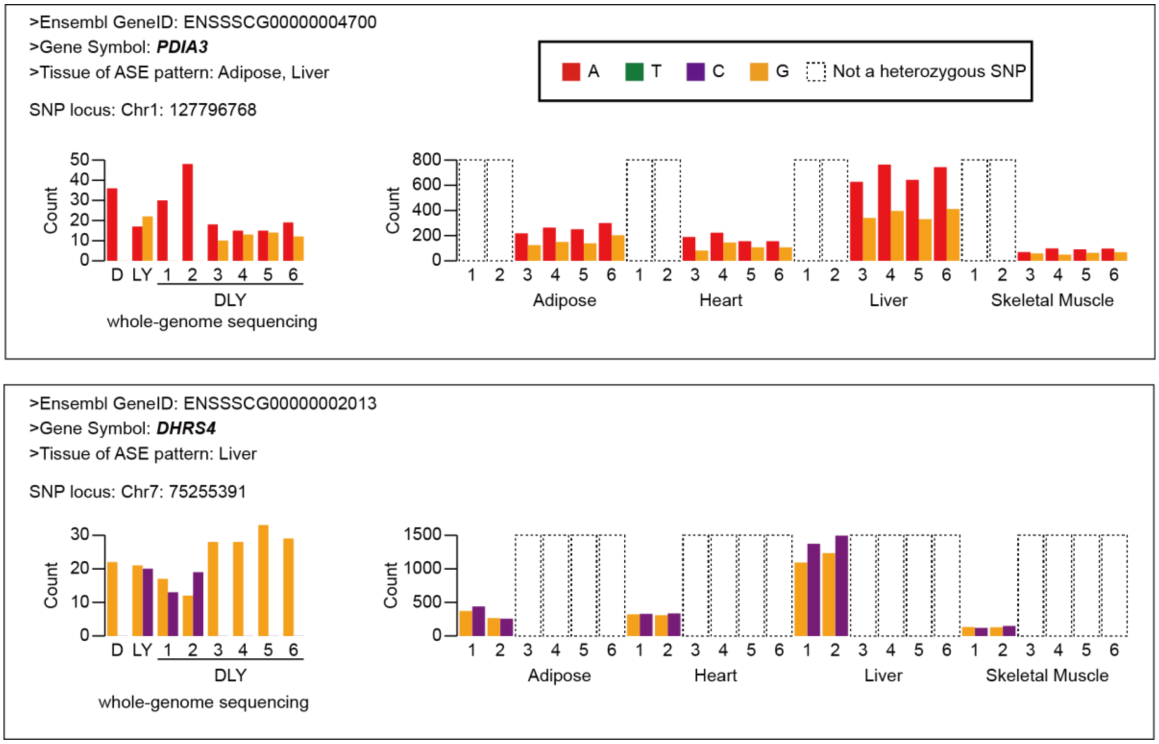

Figure S7. Visualization of 50 random selected ASE genes. The detail information of the representative SNP (maximum coverage) for each ASE gene, including the count distribution of whole-genome sequencing and the count distribution of RNA-seq of adipose, heart, liver and skeletal muscle were shown. These 50 ASE genes include *NDN*, *MTURN*, *ATP5PF*, *FBLIM1*, *APOE*, *ACOT4*, *GALNT16*, *FAH*, *EXOC2*, *ABCB11*, *SPP2*, *TM4SF20*, *GGCT*, *CYP4A24*, *TCP11L2*, *TAF13*, *CBR4*, *ENSSSCG00000035617*, *CTSB*, *RPL9*, *BOD1*, *MCFD2*, *MRPL24*, *SHISA2*, *IGF2*, *NSA2*, *GPX1*, *ENSSSCG00000035223*, *SGCE*, *YWHAZ*, *GSTA1*, *FMO5*, *SOD1*, *FOLR1*, *UGT2B31*, *FAAH*, *ANPEP*, *LDHB*, *CYP2D25*, *PTGR2*, *PPFIBP1*, *ENSSSCG00000003430*, *ENSSSCG00000040109*, *CAV1*, *LSG1*, *CYCS*, *MRLC2*, *ENSSSCG00000034663*, *PDIA3* and *DHRS4*.

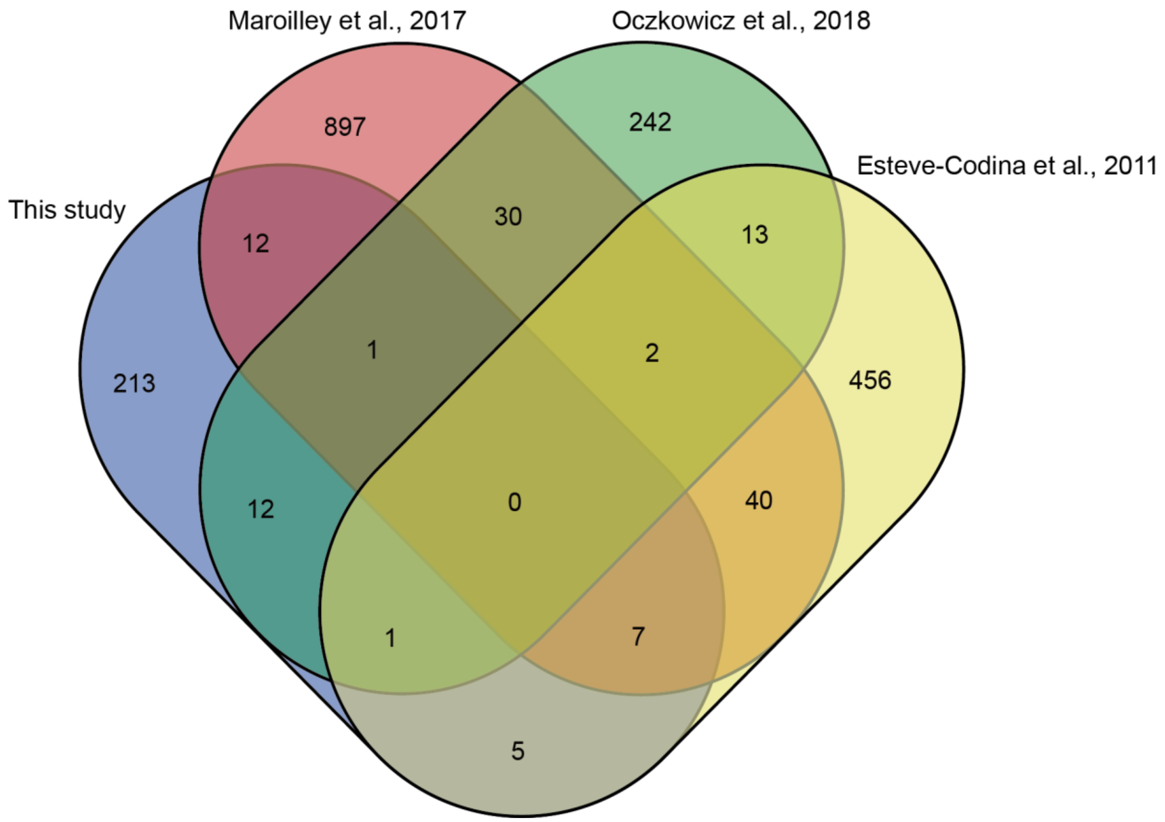

Figure S8. Comparison of identified ASE genes with previous reported studies.

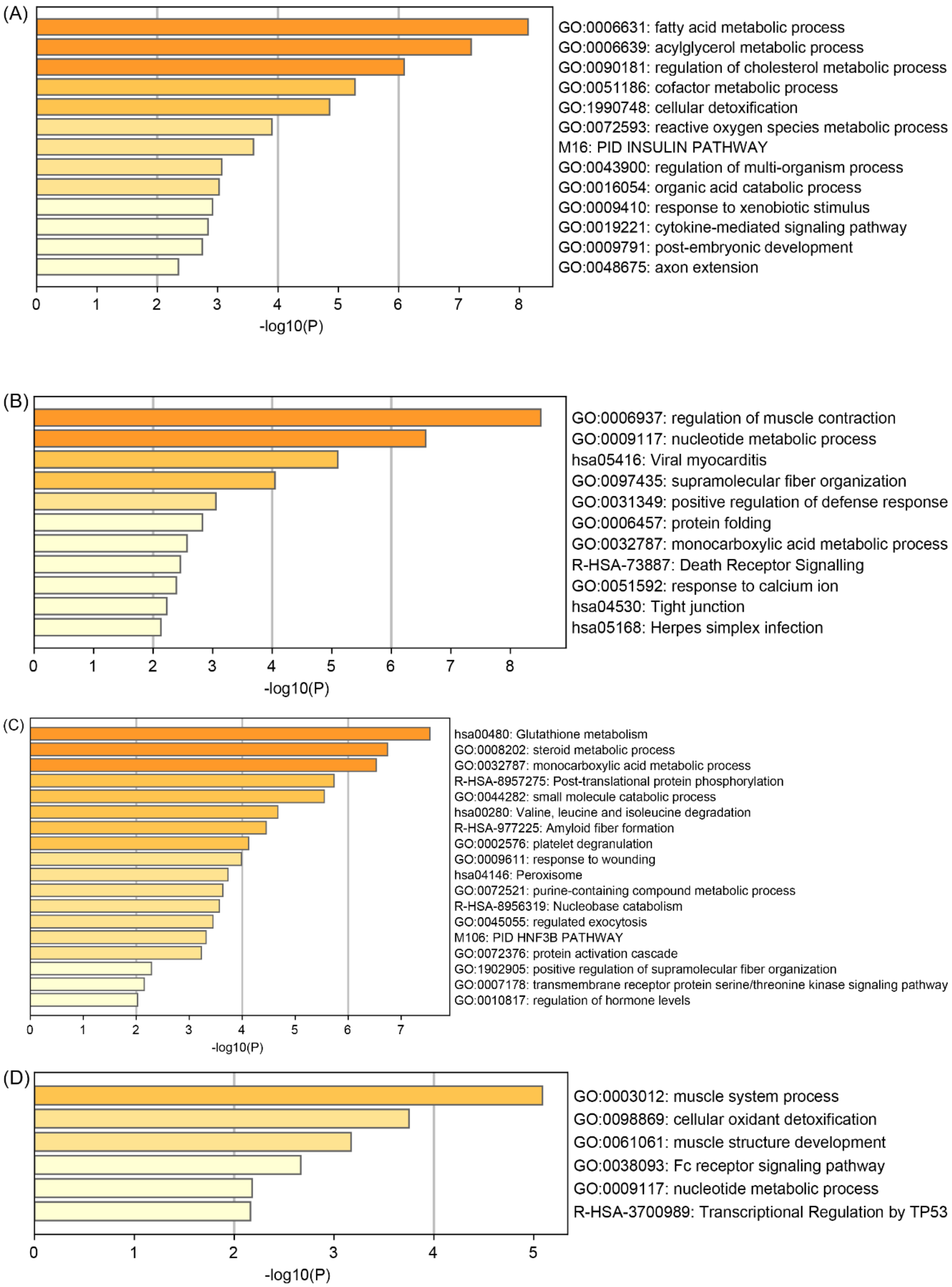

Figure S9. Bargraphs of function enrichment for ASE genes in adipose (A), heart (B), liver (C) and skeletal muscle (D).

Table S1 Data summary and SNP statistics of the downloaded pig breeds

Breed	Individual	Accession No.	High-quality data (Gb)	Coverage (×)	Number of SNPs	
					Heterozygous	Homozygous
D	1	SRX703502	38.41	15.36	2,970,948	1,970,902
	2	SRX703503	43.73	17.49	2,915,641	1,961,520
	3	SRX703538	41.63	16.65	3,017,625	1,926,350
	4	SRX703508	40.02	16.01	3,131,836	1,817,067
	5	SRX703504	43.15	17.26	2,739,820	1,947,156
	6	SRX703511	36.85	14.74	2,997,191	1,956,779
	7	SRX703509	42.92	17.17	2,788,873	1,991,953
	8	SRX703532	44.33	17.73	2,853,844	1,923,391
	9	SRX703535	35.44	14.18	2,939,385	2,032,922
	10	SRX703537	35.52	14.21	3,079,185	1,879,770
	11	SRX703539	38.35	15.34	3,045,528	1,883,315
L	1	SRX703541	35.62	14.25	3,054,680	3,089,734
	2	SRX703540	34.61	13.84	3,147,921	3,091,648
	3	SRX703542	33.61	13.44	3,074,258	3,135,548
	4	SRX703543	32.42	12.97	3,035,506	3,187,635
	5	SRX703548	33.23	13.29	3,553,960	2,825,913
	6	SRX703550	32.84	13.14	2,843,044	3,283,290
	7	SRX703552	33.25	13.30	3,818,360	2,727,436
	8	SRX703551	32.62	13.05	3,747,785	2,812,506
	9	SRX1544487	51.71	20.68	3,029,667	2,967,386
Y	1	SRX703594	31.83	12.73	2,895,003	3,365,363
	2	SRX703596	35.13	14.05	3,646,253	2,814,432
	3	SRX703599	33.42	13.37	3,462,828	3,063,392
	4	SRX703597	34.27	13.71	3,511,229	3,076,198
	5	SRX703577	44.18	17.67	3,050,148	3,261,253
	6	SRX703579	43.84	17.54	3,210,946	3,054,866
	7	SRX703581	42.33	16.93	3,226,449	3,159,891
	8	SRX703582	43.22	17.29	3,331,057	3,026,034
	9	SRX703595	34.01	13.60	3,133,195	3,047,464
	10	SRX1544506	51.63	20.65	3,649,140	2,974,150
average	-	-	38.67	15.47	-	-
Total	-	-	1,160.12	-	12,564,044	

Table S2 Data summary and SNP statistics of the DLY pig family

Breed / Crossbred	Individual	High-quality data (Gb)	Coverage (×)	Q20 (%)	Genome coverage at least			Number of SNPs	
					1× (%)	4× (%)	10× (%)	Heterozygous	Homozygous
D	1	86.16	34.44	96.52	99.53	99.27	98.19	4,437,816	2,165,490
L	1	108.22	43.28	96.64	99.45	99.13	98.38	4,722,522	3,744,240
Y	1	90.43	36.16	95.58	99.16	98.85	98.01	5,444,996	3,399,276
LY	1	90.83	36.32	95.13	99.12	98.77	97.57	5,617,905	3,335,535
DLY	1	102.11	40.84	95.53	99.57	99.33	98.68	6,263,658	2,196,226
	2	98.62	39.44	96.36	99.57	99.34	98.68	6,163,204	2,206,296
	3	97.54	39.00	95.65	99.57	99.32	98.61	6,135,372	2,178,981
	4	94.87	37.92	96.51	99.19	98.97	98.45	6,115,540	2,208,917
	5	94.56	37.80	96.09	99.19	98.96	98.45	6,167,311	2,175,091
	6	99.12	39.64	96.31	99.21	98.98	98.48	6,201,672	2,243,369
Average	-	96.25	38.51	96.03	99.36	99.09	98.35	-	-
Total	-	962.46	-	-	-	-	-	13,288,608	

Table S3 Validation of the accuracy of SNP identification by the chip

SNP type	Category	D	L	Y	LY	DLY					
						1	2	3	4	5	6
Homozygous	SNPs identified by the chip	4,323	8,740	7,560	7,698	4,586	4,742	4,464	4,447	4,645	4,549
	Validated by GATK	4,210	8,591	7,429	7,584	4,485	4,655	4,390	4,365	4,553	4,465
	Concordance ratio (%)	97.39	98.30	98.27	98.52	97.80	98.17	98.34	98.16	98.02	98.15
Heterozygous	SNPs identified by the chip	11,422	11,552	14,223	13,870	15,843	15,484	15,768	15,766	15,610	16,033
	Validated by GATK	11,254	11,303	13,873	13,664	15,613	15,243	15,527	15,528	15,390	15,814
	Concordance ratio (%)	98.53	97.84	97.53	98.51	98.55	98.44	98.47	98.49	98.59	98.63
Total	SNPs identified by the chip	15,745	20,292	21,783	21,568	20429	20,226	20,232	20,213	20,255	20,582
	Validated by GATK	15,464	19,894	21,300	21,248	20098	19,898	19,917	19,893	19,943	20,279
	Concordance ratio (%)	98.22	98.04	97.78	98.52	98.38	98.38	98.44	98.42	98.46	98.53

Table S4 Function enrichment of ASE genes in adipose

Term	Description	Count	%	Log10(P)	Log10(q)
GO:0006631	fatty acid metabolic process	11	16.67	-8.14	-3.83
GO:0006639	acylglycerol metabolic process	7	10.61	-7.20	-3.46
GO:0090181	regulation of cholesterol metabolic process	5	7.58	-6.09	-2.68
GO:0051186	cofactor metabolic process	10	15.15	-5.27	-1.96
GO:1990748	cellular detoxification	5	7.58	-4.85	-1.78
GO:0072593	reactive oxygen species metabolic process	6	9.09	-3.90	-1.24
M16	PID INSULIN PATHWAY	3	4.55	-3.59	-1.07
GO:0043900	regulation of multi-organism process	6	9.09	-3.07	-0.73
GO:0016054	organic acid catabolic process	5	7.58	-3.02	-0.70
GO:0009410	response to xenobiotic stimulus	5	7.58	-2.91	-0.60
GO:0019221	cytokine-mediated signaling pathway	8	12.12	-2.84	-0.54
GO:0009791	post-embryonic development	3	4.55	-2.74	-0.47
GO:0048675	axon extension	3	4.55	-2.35	-0.17

Table S5 Function enrichment of ASE genes in heart

Term	Description	Count	%	Log10(P)	Log10(q)
GO:0006937	regulation of muscle contraction	8	15.38	-8.51	-4.35
GO:0009117	nucleotide metabolic process	11	21.15	-6.58	-3.34
hsa05416	Viral myocarditis	4	7.69	-5.10	-2.27
GO:0097435	supramolecular fiber organization	8	15.38	-4.05	-1.50
GO:0031349	positive regulation of defense response	6	11.54	-3.05	-0.71
GO:0006457	protein folding	4	7.69	-2.83	-0.54
GO:0032787	monocarboxylic acid metabolic process	6	11.54	-2.57	-0.30
R-HSA-73887	Death Receptor Signalling	3	5.77	-2.46	-0.21
GO:0051592	response to calcium ion	3	5.77	-2.39	-0.17
hsa04530	Tight junction	3	5.77	-2.23	-0.05
hsa05168	Herpes simplex infection	3	5.77	-2.13	0.00

Table S6 Function enrichment of ASE genes in liver

Term	Description	Count	%	Log10(P)	Log10(q)
hsa00480	Glutathione metabolism	6	7.32	-7.54	-3.22
GO:0008202	steroid metabolic process	10	12.20	-6.74	-2.90
GO:0032787	monocarboxylic acid metabolic process	13	15.85	-6.53	-2.88
R-HAS 8957275	Post-translational protein phosphorylation	6	7.32	-5.73	-2.27
GO:0044282	small molecule catabolic process	10	12.20	-5.55	-2.27
hsa00280	Valine, leucine and isoleucine degradation	4	4.88	-4.67	-1.71
R-HSA-977225	Amyloid fiber formation	5	6.10	-4.45	-1.63
GO:0002576	platelet degranulation	5	6.10	-4.12	-1.41
GO:0009611	response to wounding	10	12.20	-3.99	-1.34
hsa04146	Peroxisome	4	4.88	-3.73	-1.23
GO:0072521	purine-containing compound metabolic process	9	10.98	-3.63	-1.16
R-HSA-8956319	Nucleobase catabolism	3	3.66	-3.57	-1.14
GO:0045055	regulated exocytosis	10	12.20	-3.45	-1.05
M106	PID HNF3B PATHWAY	3	3.66	-3.32	-0.97
GO:0072376	protein activation cascade	5	6.10	-3.23	-.092
GO:1902905	positive regulation of supramolecular fiber organization	4	4.88	-2.29	-0.20
GO:0007178	transmembrane receptor protein serine/threonine kinase signaling pathway	5	6.10	-2.15	-0.13
GO:0010817	regulation of hormone levels	6	7.32	-2.02	-0.04

Table S7 Function enrichment of ASE genes in skeletal muscle

Term	Description	Count	%	Log10(P)	Log10(q)
GO:0003012	muscle system process	6	23.08	-5.09	-0.77
GO:0098869	cellular oxidant detoxification	3	11.54	-3.75	0.00
GO:0061061	muscle structure development	5	19.23	-3.17	0.00
GO:0038093	Fc receptor signaling pathway	3	11.54	-2.67	0.00
GO:0009117	nucleotide metabolic process	4	15.38	-2.18	0.00
R-HSA-3700989	Transcriptional Regulation by TP53	3	11.54	-2.16	0.00
