# Supplementary material for: Genomic analyses provide insights into breed-of-origin effects from purebreds on three-way crossbred pigs
Source: PeerJ. 2019 Nov 11;7:e8009. doi: 10.7717/peerj.8009 (PMC6855203; doi:10.7717/peerj.8009)
Supplement: Supplemental Information 2 [file peerj-07-8009-s002.docx]

**MIAME Checklist**

**Experiment Design**

Goal: To confirm the accuracy of SNPs identified by whole-genome sequencing, we performed the porcine 60K BeadChip genotyping array (Illumina) for ten pig individuals of a DLY pig family.

Brief description: Crossbreeding has been an effective method to improve crossbred performance in pig industry. To have a global view of a classic three-way crossbreeding system of Duroc × (Landrace × Yorkshire) (DLY), we identified SNPs for each pig breed and crossbred individual originated from a DLY pig family to estimate the influence of purebreds on crossbred offspring using whole-genome sequencing. To confirm the accuracy of the SNPs identified by whole-genome sequencing, therefore, we performed the porcine 60K BeadChip genotyping array (Illumina) for each sequenced pig individual.

Keywords: Crossbreeding, DLY, SNP

Links: Raw data of ten Microarray experiments were submitted to the Gene Expression Omnibus database (http://www.ncbi.nlm.nih.gov/geo). The platform is GPL22098. The accession ID is GSE123327.

**Samples used**

The ear and liver tissue of ten pig individuals were used to perform Microarray experiments.

Sample Tissue

D ear

L ear

Y ear

LY ear

DLY-1 liver

DLY-2 liver

DLY-3 liver

DLY-4 liver

DLY-5 liver

DLY-6 liver

**Measurement Data and Specifications**

Genomic DNA for each individual was extracted from the ear and liver tissue using Qiagen DNeasy Tissue kit (Qiagen, Germany). 750ng of genomic DNA was whole-genome amplified in an overnight reaction at 37℃ using amplification master mix (WG-AMM) and primer/neutralization mix (WG-MP1). After incubation the amplified DNA was fragmented with fragmentation mix (WG-FRG), precipitated with isopropanol and precipitation mix (PA1) and resuspended in hybridization buffer (RA1). Resuspended DNA was loaded onto BeadChips arrays. After overnight incubation at 48℃, single-base extension and allele-specific staining was performed on a Teflow chamber rack system (Tecan, Maennedorf, Switzerland). After allele-specific staining BeadChip arrays were coated with XC4/ethanol , dried for 1 hour and scanned on iScan (Illumina Inc.)(Illumina).

**Array Design**

Commercially available porcine 60K BeadChip genotyping array (Illumina).
